# Editorial: A year in review: discussions in RNA

**DOI:** 10.3389/fgene.2025.1752525

**Published:** 2025-12-16

**Authors:** Sybille Krauß, Nicoletta Potenza

**Affiliations:** 1 Human Biology/Neurobiology, Institute of Biology, Faculty IV: School of Science and Technology, University of Siegen, Siegen, Germany; 2 Department of Environmental, Biological and Pharmaceutical Sciences and Technologies, University of Campania “Luigi Vanvitelli”, Caserta, Italy

**Keywords:** RNA-binding protein, RNA granules, microRNAs, neurodegeneration, schizophrenia, long non-coding RNA, cancer, mitochondrial permeability transition

Over the last two decades, there has been a tremendous expansion in our understanding of RNA biology. A plethora of novel RNA biotypes has been discovered, and it is now increasingly clear that RNA molecules, beyond serving as a carrier of genetic information between DNA and proteins, play pivotal roles in the control of gene expression, by adding multilayered regulatory complexity. In fact, their structural diversity and complexity are reflected in great functional versatility, which makes them capable of interacting with other RNA molecules, DNA, and proteins to regulate key biological pathways, both in physiological and pathological contexts.

This Research Topic aimed to stimulate discussions in the continuously evolving field of RNA research, bringing together four articles that offer novel research tools and mechanistic insights along with translational opportunities contributing to scientific advances in RNA research.

Filcenkova et al. address a common challenge in the field of RNA–protein interaction, namely, the lack of an easy-to-perform, rapid, and robust method to detect RNA–protein binding. The authors used the RNA-binding protein HuR (human antigen R) that binds to its target mRNAs via an AU-rich element as an example to establish a novel read-out system. In this system, a split luciferase reporter was used. One luciferase fragment was expressed as a fusion protein with HuR. The second streptavidin-coated luciferase fragment was coupled to a biotinylated RNA-oligo comprising an AU-rich HuR-binding element. The binding between HuR and its target RNA-oligo then led to reconstitution of the functional luciferase that can be detected via luminescence. The authors then used this assay to screen a set of small molecules targeting the HuR–RNA interaction, demonstrating that this novel read-out technique can be used for compound screening. Since RNA–protein interactions play an important regulatory role in several biological processes, this technique will be useful not only for studying the HuR–RNA interaction but for many other applications.

Given the association between RNAs and their interacting proteins, the subcellular localization of RNA is a critical aspect of gene expression regulation. RNA transcripts are not randomly distributed in the cell but localize to specific subcellular compartments to carry out their timely and locally regulated function. Reisbitzer et al. summarize in their perspective article how RNA is transported. Mechanisms discussed in this article include passive diffusion, phase separation into RNA granules, and active transport. The specific intracellular localization of RNA is critical for its function and stability. For example, RNA transport into membrane-less RNA granules via liquid–liquid phase separation can lead to translational block (e.g., in stress granules) or to RNA degradation (e.g., in processing bodies). Moreover, regulatory factors that promote cellular transport ranging from RNA-binding proteins to regulatory sequence elements within the RNA molecule are summarized. Importantly, all these transport mechanisms not only support the physiological function of the cell, but their deregulation also contributes to disease development. As an example for such deregulation, the mislocalization of RNA in neurodegenerative diseases is discussed.

Schizophrenia is another nervous system disease in which RNAs have emerged as critical regulators. Mohamed et al. discuss the role of single-nucleotide polymorphisms (SNPs) and microRNAs (miRNAs) in the pathogenesis of schizophrenia. The review summarizes SNPs in schizophrenia risk genes affecting the dopaminergic, glutamatergic, and GABAergic systems. Furthermore, the review addresses the potential of SNPs to affect the interaction between miRNAs and their target mRNAs. Such differences in the miRNA–mRNA interaction lead to deregulation of schizophrenia risk genes and thus influence disease susceptibility. Examples of SNPs that affect the likelihood of schizophrenia development include polymorphisms in genes involved in miRNA processing, namely, *DICER*, *DROSHA*, and *DGCR8*. Knowledge about SNPs within miRNAs that affect the expression of disease-relevant genes is important to deepen our understanding of pathogenic mechanisms.

miRNAs represent one important group of non-coding RNAs acting as post-transcriptional “micromanager” of gene expression; long non-coding RNAs (lncRNAs) are another group of RNAs that is increasingly attracting attention because of their role in gene expression regulation at multiple levels, from transcriptional to post-transcriptional control. Lin et al. demonstrate the biomarker potentiality of lncRNAs involved in the mitochondrial permeability transition (MPT) for the prognosis and therapy of breast cancer (BC), whose treatment and predictive prognosis are still challenging, due to its heterogeneous nature. The rationale of the study is based on the following evidence: lncRNAs play a pivotal role in the onset and progression of BC and may serve as a diagnostic/prognostic tool; recently, MPT has been studied for its prognostic value and potential as a therapeutic target in cancer; additionally, some studies identified the elusive connection between MPT and lncRNAs. In the article, a clinical prediction model was created by incorporating clinical variables and the MPT-related lncRNA score (MPTRscore), using LASSO–Cox regression on bulk RNA-seq data of MPT-related lncRNAs acquired from TCGA and GTEx projects. The analyses revealed two MPTRscore groups characterized by different biomolecular processes, tumor microenvironment (TME) patterns, immunotherapy/chemotherapy responses, and clinical outcomes. MPTRscore was found to rely on the hub lncRNA transcript RP11-573D15.8-018, whose oncogenic role was experimentally validated *in vitro*. Overall, the study presents a robust, mitochondria-centered lncRNA signature (MPTRscore) as a novel biomarker for BC prognosis, subtype classification, and therapeutic response.

Overall, the articles featured in this Research Topic illustrate the remarkable breadth and depth of contemporary RNA research, spanning from the development of versatile tools to study RNA–protein interactions to emerging concepts in RNA localization and transport, to the impact of genetic variation on RNA-mediated regulation in neuropsychiatric disorders, and finally to the identification of lncRNA signatures with prognostic and therapeutic relevance in cancer. These contributions collectively highlight the central role of RNA as a dynamic regulator of cellular function. As the field continues to evolve, integrative approaches combining biochemical, molecular, computational, and clinical perspectives will be essential to fully unravel the multifaceted roles of RNA and to harness this knowledge for diagnostic and therapeutic innovation, including in the context of precision medicine.

